# Genetic variation in *CFH* predicts phenytoin-induced maculopapular exanthema in European-descent patients

**DOI:** 10.1212/WNL.0000000000004853

**Published:** 2018-01-23

**Authors:** Mark McCormack, Hongsheng Gui, Andrés Ingason, Doug Speed, Galen E.B. Wright, Eunice J. Zhang, Rodrigo Secolin, Clarissa Yasuda, Maxwell Kwok, Stefan Wolking, Felicitas Becker, Sarah Rau, Andreja Avbersek, Kristin Heggeli, Costin Leu, Chantal Depondt, Graeme J. Sills, Anthony G. Marson, Pauls Auce, Martin J. Brodie, Ben Francis, Michael R. Johnson, Bobby P.C. Koeleman, Pasquale Striano, Antonietta Coppola, Federico Zara, Wolfram S. Kunz, Josemir W. Sander, Holger Lerche, Karl Martin Klein, Sarah Weckhuysen, Martin Krenn, Lárus J. Gudmundsson, Kári Stefánsson, Roland Krause, Neil Shear, Colin J.D. Ross, Norman Delanty, for the EPIGEN Consortium;, Munir Pirmohamed, Bruce C. Carleton, for the Canadian Pharmacogenomics Network for Drug Safety;, Fernando Cendes, Iscia Lopes-Cendes, Wei-ping Liao, Terence J. O'Brien, Sanjay M. Sisodiya, for the EpiPGX Consortium;, Stacey Cherny, Patrick Kwan, Larry Baum, for the International League Against Epilepsy Consortium on Complex Epilepsies;, Gianpiero L. Cavalleri

**Affiliations:** Author affiliations are provided at the end of the article.

## Abstract

**Objective:**

To characterize, among European and Han Chinese populations, the genetic predictors of maculopapular exanthema (MPE), a cutaneous adverse drug reaction common to antiepileptic drugs.

**Methods:**

We conducted a case-control genome-wide association study of autosomal genotypes, including Class I and II human leukocyte antigen (HLA) alleles, in 323 cases and 1,321 drug-tolerant controls from epilepsy cohorts of northern European and Han Chinese descent. Results from each cohort were meta-analyzed.

**Results:**

We report an association between a rare variant in the complement factor H–related 4 (*CFHR4*) gene and phenytoin-induced MPE in Europeans (*p* = 4.5 × 10^–11^; odds ratio [95% confidence interval] 7 [3.2–16]). This variant is in complete linkage disequilibrium with a missense variant (N1050Y) in the complement factor H (*CFH*) gene. In addition, our results reinforce the association between HLA-A*31:01 and carbamazepine hypersensitivity. We did not identify significant genetic associations with MPE among Han Chinese patients.

**Conclusions:**

The identification of genetic predictors of MPE in CFHR4 and CFH, members of the complement factor H–related protein family, suggest a new link between regulation of the complement system alternative pathway and phenytoin-induced hypersensitivity in European-ancestral patients.

Idiosyncratic cutaneous adverse drug reactions (cADRs) can have a genetic predisposition. The HLA-B*15:02 allele is a predictor of carbamazepine-induced Stevens-Johnson syndrome and toxic epidermal necrolysis (SJS/TEN) in individuals of Han Chinese and Southeast Asian descent, while a recent meta-analysis suggests that the allele is also a significant risk factor for oxcarbazepine-, phenytoin-, and lamotrigine-induced SJS/TEN.^1,2^ However, the association with HLA-B*15:02 does not extend to the milder but more common maculopapular exanthema (MPE) phenotype, and the allele is specific to individuals of Asian descent, limiting clinical utility across populations.^3,4^ HLA-A*31:01 has been confirmed as a transethnic risk factor for carbamazepine-induced cADRs, with the allele observed across populations of European, Japanese, and Korean descent.^5–8^ Recently, HLA-A*24:02 has been shown to associate with SJS in Han Chinese patients, irrespective of causal drug studied.^9^ Genetic variation beyond the major histocompatibility locus has also been associated with cADRs. The CYP2C9*3 allele correlates with phenytoin hypersensitivity in Han Chinese from Taiwan,^10^ with a similar effect reported in a Thai population.^11^ However, a genome-wide association study (GWAS) of lamotrigine and phenytoin-induced cADRs in Europeans did not detect significant predictors.^12^ A summary of the associated genetic risk variants for cADRs in various populations is provided in table e-1 (links.lww.com/WNL/A56).

The EpiPGX Consortium was established to identify genetic markers of epilepsy treatment response. The International League Against Epilepsy Complex Genetics Consortium (ILAE-CGC) facilitates the discovery of genetic variants influencing epilepsy predisposition.^13^ The EPIGEN consortium is a worldwide epilepsy genetics research framework and the Canadian Pharmacogenomics Network for Drug Safety (CPNDS) is an active surveillance network focused on identifying genomic markers of severe adverse drug reactions (ADRs) in children and adults.^14^ Collaboration among these consortia has provided detailed phenotypes and genotypes for over 15,000 epilepsy cases, for the investigation of antiepileptic drug (AED)–induced MPE.

Availing of the joint resources of EpiPGX, ILAE-CGC, EPIGEN, and CPNDS, this study aimed to characterize, among European and Han Chinese populations, the genetic predictors of MPE, a cutaneous ADR common to particular AEDs. Specifically, we set out to test the following hypotheses: (1) whether population-specific genetic variants predict MPE; (2) whether transethnic genetic variants predict MPE; (3) whether population-specific genetic variants predict AED-specific MPE; and (4) whether transethnic genetic variants predict AED-specific MPE.

## Methods

### Standard protocol approvals, registrations, and patient consents

All study participants provided written, informed consent for genetic analysis. Local institutional review boards approved study protocols at each contributing site.

### Study design

We conducted a retrospective case-control study in individuals of European and Han Chinese ethnicity. Participants were exposed to carbamazepine, lamotrigine, phenytoin, or oxcarbazepine. Our analyses were structured to test genetic variants for association with MPE within and across both of the broad ancestral groups, through logistic regression of genotype dosage and subsequent meta-analysis of regression coefficients. We tested for association with (1) aromatic AED-induced MPE vs controls tolerant to at least 3 aromatic AEDs, (2) carbamazepine-induced MPE vs carbamazepine-tolerant controls, (3) lamotrigine-induced MPE vs lamotrigine-tolerant controls, and (4) phenytoin-induced MPE vs phenytoin-tolerant controls. Due to small sample size, oxcarbazepine-related MPE was not analyzed as an individual case cohort.

### Cohorts and phenotype definition

Epilepsy cohorts from the ILAE-CGC, EpiPGX, and EPIGEN Consortia were included in the discovery GWAS meta-analysis (table 1). A European-descent replication cohort was assembled from sites in Brazil, Canada (via CPNDS), Liverpool, and other sites across the United Kingdom. Cases were defined as having MPE attributed to carbamazepine, lamotrigine, phenytoin, or oxcarbazepine as determined by their clinician, occurring within 3 months of initiation and resolving upon dose reduction or AED withdrawal. Control patients trialed carbamazepine, lamotrigine, phenytoin, or oxcarbazepine for at least 3 months without reporting a cADR. Epilepsy-specific patient demographics are presented in table e-2 (links.lww.com/WNL/A56).

**Table 1:**
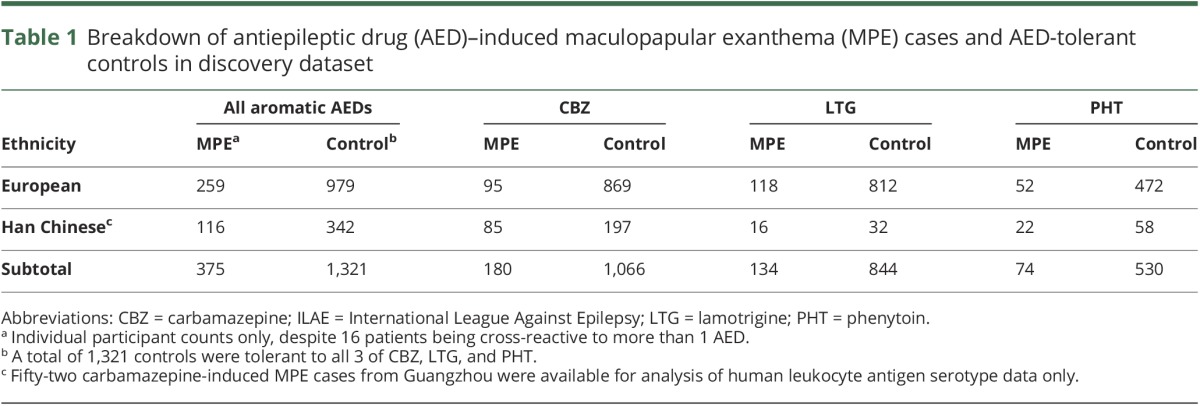
Breakdown of antiepileptic drug (AED)–induced maculopapular exanthema (MPE) cases and AED-tolerant controls in discovery dataset

### Genotyping and imputation

Genotyping of a subset of EpiPGX samples was performed at deCODE Genetics (Reykjavik, Iceland) using Illumina (San Diego, CA) OmniExpress-12 v1.1 and OmniExpress-24 v1.1 single nucleotide polymorphism (SNP) arrays. The remainder of samples were genotyped locally using various Illumina beadchip SNP arrays, details of which are published elsewhere.^13^ Genotyping and imputation quality control is described in appendix e-1 (links.lww.com/WNL/A57).

### Study power

We estimated that the study had 80% power to detect a genetic predictor of relative risk (approximated to odds ratio) ≥3 with an allele frequency ≥2% and an α level of 1.25 × 10^−8^. Power for AED-specific and population-specific analyses are detailed in appendix e-1 (links.lww.com/WNL/A57).

### Statistical analyses

Association analyses were conducted within the European and Asian subgroups using an additive logistic regression model. To account for genotype uncertainty, SNPTEST was used to apply a missing data likelihood score model that included sex, clinical site, and 5 principal components as covariates to control for bias and population stratification.^15^ Fixed effects meta-analyses were conducted across the European and Asian subgroups using the software package METAL, applying genomic control correction within cohorts.^16^ The threshold for statistical significance was set at 1.25 × 10^−8^, reflecting an empirical Bonferroni correction for 4 tests, of the standard 5 × 10^−8^ genome-wide significance threshold. Conditional association analysis was performed on loci containing significant markers to establish whether other genetic variants in the region (1 Mb upstream and downstream) were independently associated with MPE. The conditional threshold for significance was set at 5 × 10^−6^, based on a genome-wide estimation of 10,000 imputed variants per 2 MB region.^13^ We applied the Stouffer *z* trend test to the combined results from the discovery and replication cohorts.

### Confirmatory genotyping

Where an association signal satisfied the threshold for significance, additional genotyping and resequencing were performed in a subset of patients and results were compared with imputation dosage files. The variant rs78239784 was confirmed by Sanger sequencing in 100 patients from the original discovery cohort. For the purpose of replication, we genotyped the rs78239784 variant in an independent cohort of 13 phenytoin-induced MPE cases and 88 phenytoin-tolerant controls.

## Results

### Cohort description

In total, 375 MPE cases and 1,321 controls satisfied our criteria for inclusion in the discovery analyses (see Methods and table 1). There were 16 patients with cross-reactivity to 2 or more aromatic AEDs, 8 of whom were hypersensitive to carbamazepine and lamotrigine. Genome-wide array data for 323 cases and 1,321 controls were available for analysis. Broad European or Han Chinese ancestry was assigned to each participant according to principal components analysis (figure e-1, links.lww.com/WNL/A55).

### Genome-wide association analysis of broad aromatic AED–induced MPE

After quality control (see appendix e-1, figure e-2, links.lww.com/WNL/A55, for details), 3,693,290 variants remained for analysis in the European dataset and 4,402,554 variants in the Han Chinese dataset. We only considered autosomal SNPs in our analyses. To test hypothesis (1), that population-specific genetic markers predispose to MPE, a logistic regression analysis of all MPE cases was performed separately in the European and Han Chinese ancestral subgroups. We did not observe any genome-wide significant markers for MPE due to any aromatic AED in either Europeans or Han Chinese. The study was powered to detect an effect of relative risk >3.5 in the European cohort and >5 in the Han Chinese.

**Figure 1:**
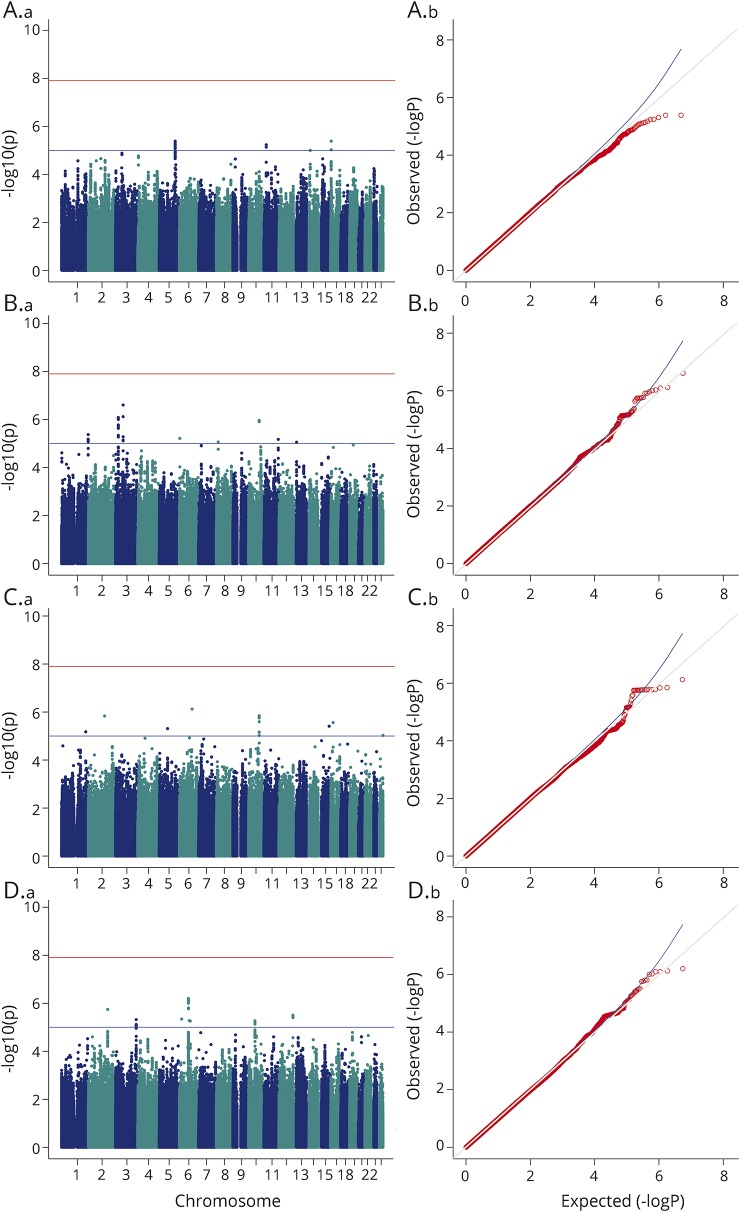
Meta-analysis results across European and Han Chinese cohorts Manhattan (a) and quantile–quantile (b) plots for the meta-analyses of maculopapular exanthema vs tolerant controls, for (A) any antiepileptic drug (genomic inflation factor [λ] = 1.01), (B) carbamazepine (λ = 1.01), (C) lamotrigine (λ = 0.99), and (D) phenytoin (λ = 0.98).

To test hypothesis (2), that transethnic genetic markers predispose to MPE, a fixed-effects meta-analysis of the association results for European and Han Chinese ancestral subgroups was performed. We did not observe any genome-wide significant markers for MPE shared among European or Han Chinese subgroups (figure 1A). This analysis was powered to detect an effect size >3.

### Genome-wide association analysis of specific aromatic AED–induced MPE

To test hypothesis (3), that genetic variants for MPE are AED-specific and population-specific, logistic regression analyses of AED-specific MPE was performed separately in the European and Han Chinese ancestral groups (figures e-3 and e-4, links.lww.com/WNL/A55). Within the European subgroup, HLA-A*31:01 was significantly associated with carbamazepine-induced MPE in Europeans (*p* = 1.47 × 10^−10^, odds ratio [OR] [95% confidence interval (CI)] 5.5 [3.0–10]). Conditioning on HLA-A*31:01 did not reveal additional variants within the human leukocyte antigen (HLA) region that were independently contributing to carbamazepine-induced MPE. No genome-wide significant signals for lamotrigine-induced MPE were observed in Europeans. This analysis was powered to detect an effect size >6.

**Figure 2:**
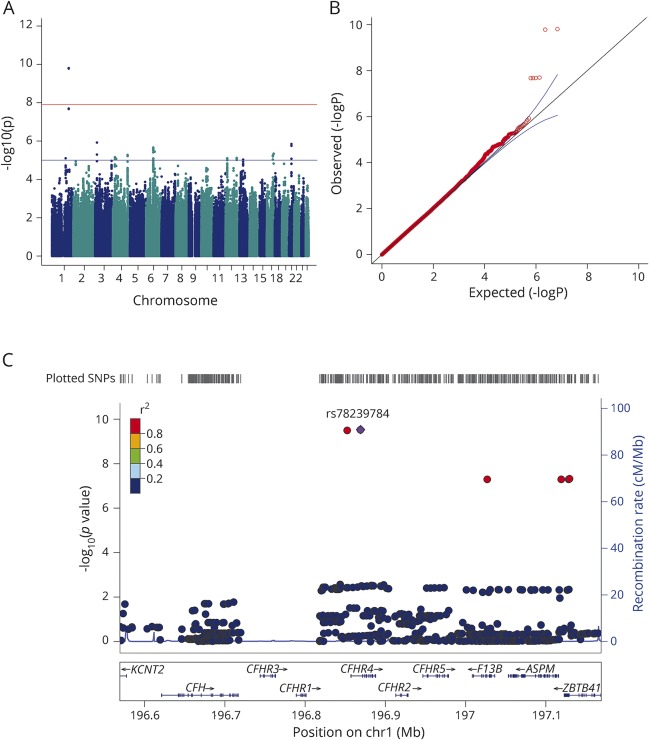
Intronic *CFHR4* variants are associated with phenytoin-induced maculopapular exanthema (MPE) in Europeans (A) Manhattan and (B) quantile–quantile plot of phenytoin-induced MPE in the European subgroup (λ = 1.01). The LocusZoom plot (C) highlights the most significant single nucleotide polymorphism (SNP), rs78239784 (purple dot), is an intronic variant in *CFHR4*.

For phenytoin-induced MPE, we identified a significant association with rs78239784, an intronic variant of the complement factor H–related 4 gene (*CFHR4*). The risk allele, G, had a minor allele frequency of 12% in our European phenytoin-induced MPE cases compared to 1.5% in European phenytoin-tolerant controls (*p* = 2.94 × 10^−10^, OR [95% CI] 8.8 [4.0–19]; figure 2). Conditioning on rs78239784 did not reveal additional variants in this locus that were independently contributing to phenytoin-induced MPE. Using 1000 Genomes Phase III European population data, rs78239784 was found to be in complete linkage disequilibrium with rs35274867 (*r*^2^ = 1 and D' = 1), a missense variant coding for an asparagine to tyrosine substitution at amino acid 1,050 of the complement factor H (*CFH*) gene. The missense variant was not present in our association results, as it was filtered during quality control because the imputation score was <0.95. The imputation accuracy of the top variant rs78239784 was confirmed in our cohort via Sanger sequencing and TaqMan approaches. Of 100 samples tested, a 100% concordance rate was found between imputed and resequenced genotypes. Within the Han Chinese subgroup, no significant associations were found between autosomal SNPs or *HLA* alleles and AED-specific MPE. Summary results for known risk loci in our dataset were scrutinized and are presented in table 2. None of these loci was even nominally significant (*p* > 0.05) in the Han Chinese subgroup.

**Table 2:**
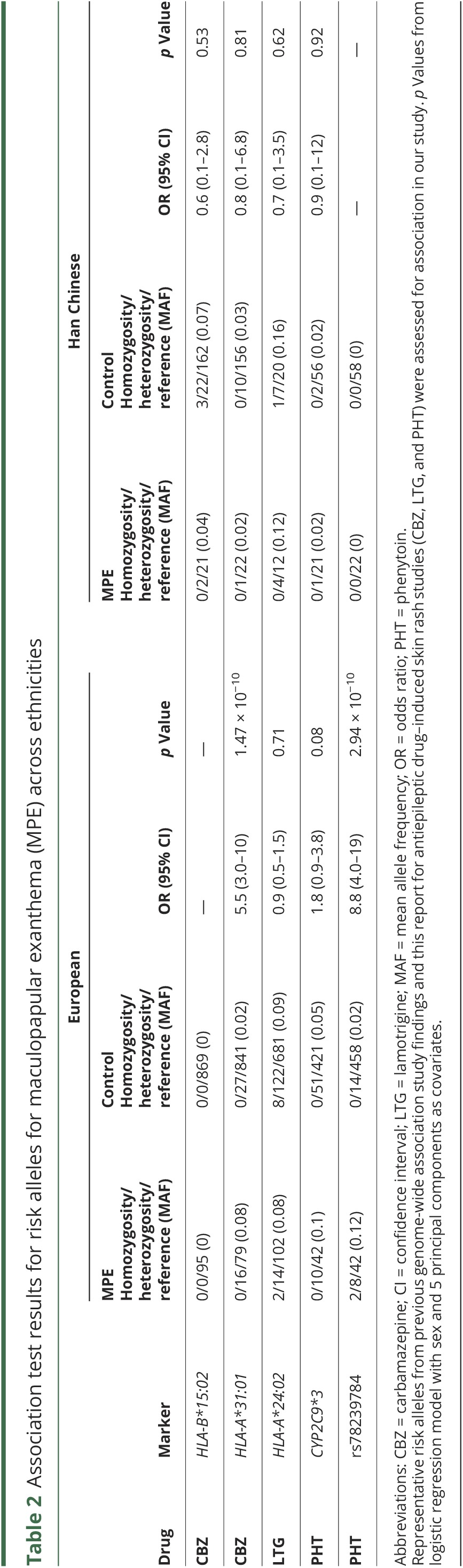
Association test results for risk alleles for maculopapular exanthema (MPE) across ethnicities

In order to test hypothesis (4), that transethnic genetic markers predispose to AED-specific MPE, we meta-analyzed *p* values from association results for carbamazepine, lamotrigine, and phenytoin individually, across European and Han Chinese ancestral subgroups. There were no shared genome-wide significant markers among our meta-analyses of AED-specific MPE (figure 1, B–D).

### Replication of *CFHR4* signal

To replicate the association with phenytoin-induced MPE in an independent cohort, the variant rs78239784 was genotyped in self-reported European-descent cases and controls recruited through centers in Liverpool (United Kingdom), Sao Paolo (Brazil), and across Canada (CPNDS). Two heterozygous carriers were identified among 13 phenytoin-induced MPE cases while only a single carrier was observed among 88 phenytoin-tolerant controls yielding a 2-tailed Fisher exact *p* value of 0.044. Pooling all cases and controls together, we report an overall *p* value of 4.5 × 10^−11^ (with a combined OR [95% CI] 7 [3.2–16]) for the association between rs78239784 and phenytoin-induced MPE in Europeans (table 3).

**Table 3:**
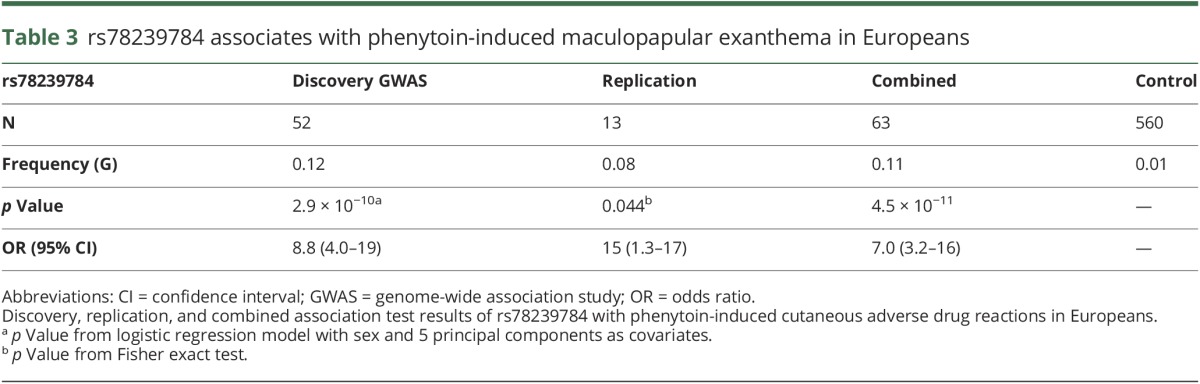
rs78239784 associates with phenytoin-induced maculopapular exanthema in Europeans

## Discussion

We detected a strong association between variants in the complement factor H regulatory pathway and phenytoin-induced MPE in a European-descent patient population. The presence of the associated genotype increases risk for MPE 6-fold. Our results indicate that risk variants for MPE tend to be drug-specific and population-specific.

These results point to the regulators of complement activation gene cluster as a genetic locus contributing to the onset of hypersensitivity to phenytoin. The most significant variant in our European subgroup analysis, rs78239784 (c.59-2448T>G), tags the missense variant rs35274867 (p.N1050Y) in *CFH*, suggesting aberrant complement activation as a potential causal mechanism in a subset of phenytoin-sensitive individuals. According to data from the Exome Aggregation Consortium, *CFH* N1050Y has an allele frequency of approximately 2% in Europeans, 3% in African subpopulations, less than 1% in South Asians, and is almost invariant in East Asians.^17^ Given the absence of this allele in East Asian populations, the lack of an association between the *CFH* locus and MPE in our Han Chinese cohort is unsurprising. We propose that population-specific, independent rare variants of large effect may explain a proportion of MPE cases, a similar paradigm to the rare variant model demonstrated in Crohn disease and ulcerative colitis.^18^ *CFH* N1050Y has previously been associated with type 2 diabetes–associated end-stage kidney disease in an African American cohort.^19^ Defects in CFH-related proteins are also associated with overactivation of the complement immune system and can lead to atypical hemolytic-uremic syndrome, C3 glomerulopathy, basal laminar drusen, immunoglobulin A nephropathies, and systemic lupus erythematosus.^20^ Further, genetic variants in *CFHR4* and *CFH* are associated with risk for age-related macular degeneration.^21^ We did not detect these symptoms among N1050Y carriers. While it is unclear whether phenytoin directly interacts with circulating CFH-related proteins, it does not specifically increase serum complement levels.^22^ Our findings offer an expanded insight into the role of the complement alternative pathway in hypersensitivity to AEDs.

Phenytoin is still used as a first-line treatment for epilepsy in many settings and is listed on the WHO list of essential medicines.^23^ Epidemiologic data on prescriptions of AEDs for epilepsy in the United Kingdom show that, in 2008, phenytoin accounted for 18% of all treated person-years in epilepsy and was most frequently used in the elderly.^24^ Therefore, a clinically useful prognostic test for phenytoin-induced cutaneous ADRs in European-ancestral individuals would be welcome. The sensitivity of the *CFHR4* variant as a prognostic marker is 16% and the specificity is 97%, which corresponds to a positive likelihood ratio of 5.93 (95% CI 2.8–12.6) and a negative likelihood ratio of 0.86 (95% CI 0.8–0.9). Assuming the pretest probability of the ADR is 5%, a positive test for this marker increases the probability of MPE to phenytoin sixfold to 30%, while a negative test reduces the probability marginally to 4.3%. There are an estimated 6 million people with epilepsy in Europe, which means approximately 90,000 people are at-risk carriers of this mutation.^25^ We estimate that 208 (95% CI 103–431) patients of European ancestry would need to be screened to prevent one case, based on a previously reported formula,^26^ which corresponds to an absolute risk reduction of 0.005 (95% CI 0.002–0.009). As a comparison, it is estimated that 442 Han Chinese patients would be needed to screened for HLA-B*15:02 in order to prevent a single carbamazepine-induced SJS/TEN case. We would suggest that the clinical utility and cost-effectiveness of implementing preemptive screening be evaluated through a prospective study.

We did not replicate the association between CYP2C9*3 (rs1057910) and MPE in our cohort, irrespective of ethnicity or AED. This is not surprising given that the original association with phenytoin in Han Chinese was largely driven by SJS/TEN cases, which were excluded from this analysis. We did, however, detect a nonsignificant enrichment of CYP2C9*3 (*p* = 0.08) among the European phenytoin-induced MPE cases, but the effect size we observe (OR 1.8) is smaller than previously reported for phenytoin-induced MPE in Han Chinese (OR 5.5).^10^ Notably, 2 CYP2C9*3 carriers among the phenytoin-induced MPE cases were also heterozygous for the *CFHR4* variant while only 3 of 560 controls were jointly heterozygous. The frequency of CYP2C9*3 differs between controls from European and Han Chinese subgroups in our study, which is in accordance with background population frequency reported in the Exome Aggregation Consortium (European: 7%, East Asian: 3%). While our results do not support a significant effect of CYP2C9*3 in MPE, larger cohorts including severe cADR cases may resolve the extent of the association across populations.

HLA-A*31:01 was the most strongly associated marker with carbamazepine-induced MPE in Europeans in this study. Forty-three of the 95 cases studied here were also included in the discovery publication,^8^ but the effect of the allele remains significant when we restrict to new cases only (p = 4 × 10^−7^), thus providing an additional independent replication of the initial finding. We confirm that HLA-B*15:02 is not associated with carbamazepine-induced MPE in Han Chinese, and no novel signals emerged for carbamazepine-induced MPE in either population. No significant predictors of lamotrigine-induced MPE were observed in either population tested. HLA-A*24:02 was not significantly associated with lamotrigine-induced MPE in either of the European or Han Chinese ancestral subgroups; rather this allele was observed to be more frequent among our lamotrigine-tolerant controls.

Our meta-analyses did not reveal any significant transethnic genetic markers for MPE due to any AED. There were considerably more European-descent patients in this analysis than any other ethnicity, and we recognize this as a limitation of the study. Analysis of non-European cohorts is warranted. A second limitation of our study was the low number of Han Chinese lamotrigine-related MPE cases, relative to European-descent cases. Therefore we cannot conclusively rule out genetic predictors of modest effect size for MPE to lamotrigine in Han Chinese or other non-European descent populations. We recognize that our replication cohort for phenytoin is small and comprises self-reported ancestral Europeans. Since we did not have full genotype array data for these individuals, we relied on Fisher exact test for calculating significance rather than logistic regression with correction for principal components. Additional studies of larger sample size are required to further characterize the association, improve the estimation of the risk effect size, and determine the prognostic ability and economics of screening for this marker. Finally, as this study was not powered to investigate MPE attributed to oxcarbazepine due to low sample size, further investigation is warranted.

We have identified a genetic predictor for a common adverse reaction to phenytoin in European-descent patients, adding a new pharmacogenetic marker for potential use in the treatment of epilepsy. This finding adds to the list of genetic predictors of hypersensitivity to anticonvulsant therapy and opens up a new avenue for understanding the biology underlying cutaneous adverse reactions. This finding can advance genetic testing in the clinic as it expands the array of genetic tests available to aid clinicians in reducing overall rates of discontinuation due to adverse events and improving patient safety.

## Affiliations

From Molecular and Cellular Therapeutics (M.M., N.D., G.L.C.) and FutureNeuro Research Centre (G.L.C.), Royal College of Surgeons in Ireland, Dublin; Centre for Genomic Sciences (H.G., S.C., L.B.), Li Ka Shing Faculty of Medicine, University of Hong Kong; deCODE Genetics/Amgen, Inc. (A.I., L.J.G., K.S.), Reykjavik, Iceland; UCL Genetics Institute (D.S.), University College London, UK; Centre for Molecular Medicine and Therapeutics, BC Children's Hospital Research Institute, Faculty of Medicine (G.E.B.W.), Departments of Pediatrics and Medical Genetics (C.J.D.R.), and Division of Translational Therapeutics, Department of Pediatrics (B.C.C.), University of British Columbia, Vancouver, Canada; Department of Molecular and Clinical Pharmacology (J.E.Z., G.J.S., A.G.M., P.A., M.P.), Institute of Translational Medicine, University of Liverpool, UK; Department of Medical Genetics (R.S., I.L.-C.), School of Medical Sciences, University of Campinas and the Brazilian Institute of Neuroscience and Neurotechnology (BRAINN); Department of Neurology (C.Y., F.C.), School of Medical Sciences, University of Campinas and the Brazilian Institute of Neuroscience and Neurotechnology (BRAINN), Campinas, SP, Brazil; Hertie Institute for Clinical Brain Research (M.K., H.L.), University of Tübingen, Germany; Department of Clinical and Experimental Epilepsy (S.W., F.B., S.R., A.A., K.H., C.L., S.M.S.) and NIHR University College London Hospitals Biomedical Research Centre (J.W.S.), UCL Institute of Neurology, London, UK; Laboratory of Experimental Neurology (C.D.), Hôpital Erasme, Université Libre de Bruxelles, Brussels, Belgium; The Walton Centre NHS Foundation Trust (A.G.M., P.A.), Liverpool; Epilepsy Unit (M.J.B.), West Glasgow ACH-Yorkhill; Department of Biostatistics (B.F.), Institute of Translational Medicine, University of Liverpool; Division of Brain Sciences (M.R.J.), Imperial College Faculty of Medicine, London, UK; Division of Neurosciences (B.P.C.K.) and Center for Molecular Medicine (M.M.), Universitair Medisch Centrum Utrecht, Netherlands; Pediatric Neurology and Muscular Diseases Unit, Department of Neurosciences, Rehabilitation, Ophthalmology, Genetics, Maternal and Child Health, University of Genoa (P.S.), and Laboratory of Neurogenetics and Neuroscience (F.Z.), Institute G. Gaslini; Department of Neuroscience (A.C.), Reproductive and Odontostomatological Sciences, Federico II University, Naples; Department of Epileptology (W.S.K.), University of Bonn, Germany; Stichting Epilepsie Instellingen Nederland (SEIN) (J.W.S.), Heemstede, Netherlands; The Chalfont Centre for Epilepsy (J.W.S., S.M.S.), Epilepsy Society, Chalfont St. Peters, Buckinghamshire, UK; Epilepsy Center Frankfurt Rhine-Main (K.M.K.), Department of Neurology, Center of Neurology and Neurosurgery, University Hospital, Goethe-University Frankfurt; Epilepsy Center Hessen (K.M.K.), Department of Neurology, University Hospitals Giessen & Marburg, and Philipps-University Marburg, Germany; Neurogenetics Group (S.W.), VIB-UAntwerp, Center for Molecular Neurology; Neurology Department (S.W.), University Hospital Antwerp, Belgium; Department of Neurology (M.K.), Medical University of Vienna, Austria; Faculty of Medicine (K.S.), University of Iceland, Reykjavik; Luxembourg Centre for Systems Biomedicine (R.K.), University of Luxembourg; University of Toronto (N.S.); Institute of Neuroscience and The Second Affiliated Hospital of Guangzhou Medical University (W.-p.L.), Key Laboratory of Neurogenetics and Channelopathies of Guangdong Province and the Ministry of Education of China, China; and Departments of Medicine and Neurology (T.J.O., P.K.), University of Melbourne, Royal Melbourne Hospital, Australia.

## Glossary

ADR: adverse drug reaction
AED: antiepileptic drug
cADR: cutaneous adverse drug reaction
*CFH*: complement factor H
*CFHR4*: complement factor H–related 4 gene
CI: confidence interval
CPNDS: Canadian Pharmacogenomics Network for Drug Safety
GWAS: genome-wide association study
HLA: human leukocyte antigen
ILAE-CGC: International League Against Epilepsy Complex Genetics Consortium
MPE: maculopapular exanthema
OR: odds ratio
SJS/TEN: Stevens-Johnson syndrome and toxic epidermal necrolysis
SNP: single nucleotide polymorphism
